# Psycho-Analysis and Other Fashions

**Published:** 1921-01-15

**Authors:** R. B. Ince

**Affiliations:** The Hermitage, Jarvis Brook, Sussex


### THE EDITOR'S LETTER-BOX.

**Correspondence on all subjects is invited, but we cannot in any way be responsible for the opinions expressed by our correspondents, who must give their name and address as a guarantee of good faith, but not necessarily for publication. Correspondents are reminded that brevity of style and conciseness of statement greatly facilitate early insertion.**

### "Psycho-Analysis and Other Fashions."

*To the Editor of* The Hospital.

Sir,—As a student of life, high, low, and middle, I have become convinced that the medical art as we have known it hitherto is a thing of the past. A great rival has sprung up by the name of Psycho-analysis. All my medical friends who have ambition are training to become psycho-analysts. And they feel convinced that there is a great future before them.

There is much to be said for psycho-analysis, and all my aforesaid friends are saying it. Consider the old method. You go to a doctor and he asks you to put out your tongue. This has always been very embarrassing to shy people. I, for one, will only put out my tongue at my doctor under protest. It is not treating the profession with respect. I would as soon make a "long nose" at my medical man as obtrude my tongue at him. And many of my friends feel the same.

Now the psycho-analyst dispenses with all that. He invites you to sit in a comfortable chair and tell him your dreams. What could be more delightful? I frequently relate my dreams to my wife at breakfast, but the woman is so unsympathetic she either doesn't listen or cuts me short with "Tscha!" or some unfeeling expletive of the kind.

The psycho-analysts assure me they can cure almost anything by the analytical method, and, despite rates and taxes, I am feeling quite exhilarated at the prospect before us. Looking a few years ahead, I see a new world from which all our difficulties will be expelled by psycho-analysis.

For instance, I see a man terribly vexed and troubled by the dread of spending money. There are many in that position to-day, and will be more to-morrow. Even to spend sixpence gives him severe pain. He consults a psycho-analyst. His dreams reveal that the trouble started when, as a child, he was made to put a penny in the collecting plate at church. He is treated, and quite recovers from the "suppressed reflex." Result: He now spends £1,000 a year without any difficulty, although only in receipt of £500 a year.

I see the case of a man who imagines he is about to die. He falls into the hands of the psycho-analysts. They discover that the origin of his morbid dread is traceable to a day when his nurse forgot to give him his bottle. Treatment is given, and now, despite the fact that he is without work and has nothing to eat, he is quite happy and lives entirely on *bona verba*.

The author of the future, vexed because his best book does not sell, consults a psycho-analyst. His dreams reveal the fact that on one occasion a publisher was very rude to him. Words were exchanged, and finally books. The scene made an impression on him that he could not shake off. Two doses of psycho-analysis cured him. He is now very rude to his publisher, and as a result his books sell. He is advertised as "the most dangerous author living."

Psycho-analysis is the science of the future. Politicians with swelled head will consult the psycho-analyst, and the swelling will be reduced. Doctors will literally fling physic to the dogs, and surgeons will have to seek work at Smithfield.

It will be a wonderful world!—Yours faithfully,

R. B. Ince.

The Hermitage, Jarvis Brook, Sussex.

